# Combined evaluation of arterial stiffness, glycemic control and hypertension for macrovascular complications in type 2 diabetes

**DOI:** 10.1186/s12933-022-01696-1

**Published:** 2022-11-28

**Authors:** Zhiyuan Wu, Siqi Yu, Haiping Zhang, Zheng Guo, Yulu Zheng, Zongkai Xu, Zhiwei Li, Xiangtong Liu, Xia Li, Shuo Chen, Jingbo Zhang, Lixin Tao, Xiuhua Guo

**Affiliations:** 1 Beijing Municipal Key Laboratory of Clinical Epidemiology, Department of Epidemiology and Health Statistics, School of Public Health, Capital Medical University, No.10 Xitoutiao, Youanmen Street, Beijing, 100069 China; 2 Centre for Precision Health, Edith Cowan University, Perth, Australia; 3 Department of Mathematics and Statistics, La Trobe University, Melbourne, Australia; 4 Beijing Physical Examination Center, Beijing, China

**Keywords:** Type 2 diabetes, Macrovascular complications, Arterial stiffness, Combined effect of arterial stiffness, glycemic control and hypertension, Multifactorial risk stratification

## Abstract

**Background:**

Arterial stiffness, glycemic control and blood pressure are risk factors of macrovascular complications in type 2 diabetes. This study aimed to investigate the combined association of arterial stiffness, glycemic control and hypertension status with the occurrence of diabetic macrovascular complication.

**Methods:**

A total of 1870 patients of diabetes were enrolled from Beijing Health Management Cohort between 2008 and 2018 as baseline, and then followed for macrovascular complication onset. We proposed a composite risk score (0–4) by arterial stiffness severity, pool glycemic control and hypertension status. Cox model was used to estimate the hazard ratio (HR) and 95% confidence interval (CI).

**Results:**

The mean age (SD) of this population was 59.90 (12.29) years. During a median follow-up of 4.0 years, 359 (19.2%) patients developed macrovascular complication. Compared to the normal arterial stiffness and good glycemic control group, patients with severe arterial stiffness and pool glycemic control had the highest risk of macrovascular complications (HR: 2.73; 95% CI: 1.42–5.25). Similarly, those of severe arterial stiffness and hypertension had the highest risk (HR: 2.69; 95% CI: 1.61–4.50). Patients of the composite score > 2 had a significantly increased risk of macrovascular complication.

**Conclusion:**

This study suggested the clinical importance of combined evaluation of arterial stiffness, glycemic control and hypertension status for the risk stratification and management of macrovascular complication of type 2 diabetes.

**Supplementary Information:**

The online version contains supplementary material available at 10.1186/s12933-022-01696-1.

## Background

Type 2 diabetes is a serious public health issue worldwide with high morbidity and mortality [1]. Diabetes increases the risk of adverse macrovascular events including coronary heart disease, cerebrovascular disease, and peripheral artery disease [2–4]. Macrovascular complications have become the major cause of mortality and impaired life quality in diabetes, leading to severe health and economic burden [5, 6].

Patients with diabetes had increased arterial stiffness level [7] and higher morbidity of cardiovascular diseases than the general population [8]. Arterial stiffness may be one possible pathway linking diabetes and increased cardiovascular risk [9]. Arterial stiffness is a predictor of cardiovascular events and mortality independent of traditional risk factors and mostly been used in research protocols. Its use as a prognostic indicator in clinical practice is still uncommon [10]. In addition, poor glycemic control and hypertension had an adverse effect on the development of diabetic macrovascular complication [11, 12]. It is known that optimal levels of blood pressure and glucose in diabetic patients are associated with substantially lower risk of cardiovascular events [13]; however, more information is needed on the value of combined multifactorial risk factor evaluation and management [14]. On the other hand, studies suggested that glycemic level and blood pressure interacted with arterial stiffness in patients with diabetes [15–17]. A recent study reported the predictive capacity of combined evaluation of arterial stiffness and hypertension status for diabetes onset [18]. However, the combined effect of arterial stiffness with glycemic control and hypertension status on macrovascular complication in the diabetic population is unknown.

Therefore, this study aimed to evaluate the combined association of arterial stiffness, glycemic control and hypertension status with the occurrence of diabetic macrovascular complication. We hypothesized that the combined assessment of arterial stiffness, glycemic control and hypertension status could improve the risk stratification and prediction of macrovascular complications in type 2 diabetes.

## Methods

### Study population

We recruited participants from the Beijing Health Management Cohort (BHMC), which is an open, dynamic and population-based cohort conducted in Beijing, China. BHMC aims to collect and investigate the biomarkers of cardiometabolic diseases including diabetes and its complications. Details of the cohort design have been described in a previous study [19].

The original cohort included 10,632 participants with physical and arterial examinations between 2008 and 2018. Then, participants without type 2 diabetes or with type 1 diabetes (n = 7495) or lacking eligible arterial stiffness data (n = 1038) were excluded at baseline. We further excluded participants with a history of macrovascular complication (n = 126), diabetic nephropathy or retinopathy (n = 71) or missing fasting glucose data (n = 32) at baseline. Finally, a total of 1 870 participants was included and followed until the development of macrovascular complication or December 31 of 2019, which came first (Additional file 1: Figure S1). All participants provided written informed consent, and this study was approved by the Ethics Committee of Capital Medical University (Grant Number: 2020SY031).

### Data collection and definition

Information on demographic characteristics and health information including age, sex, lifestyle factors (smoking status, alcohol consumption, and physical activity), and health-related data (diagnosis history of diseases and medication use) were collected using a standardized questionnaire. Smoking status and drinking status were categorized into "current" and "not current". Physical activity was defined as participating in moderate or intense exercise "≥ 20 min per time and ≥ 4 times per week". Self-reported health conditions included a physician-diagnosed history of cardiovascular diseases, diabetes, hypertension, and dyslipidemia. The use of antidiabetic, antihypertensive, and lipid-lowering medications were collected.

Physical examination includes height, weight, systolic blood pressure (SBP), and diastolic blood pressure (DBP). Body mass index (BMI) was calculated as weight (kg) divided by height^2^ (m)^2^. Obesity was defined as BMI ≥ 28 kg/m^2^ In Chinese adults [20]. Blood pressure was measured in a seated position after a rest of five minutes using a mercury sphygmomanometer, and the average value of two readings was recorded. The laboratory test data were obtained from the electronic medical record system. The data of serum total cholesterol, triglycerides, high-density lipoprotein (HDL) cholesterol, low-density lipoprotein (LDL) cholesterol, fasting glucose, serum creatinine, and urinary albumin/creatinine ratio (UACR) at baseline were used in this current study.

Hypertension was defined as SBP ≥ 140 mmHg or DBP ≥ 90 mmHg or use of any antihypertensive medication or self-reported history of hypertension according to the JNC-7 criteria [21]. The diagnosis of diabetes referred to the American Diabetes Association, including fasting glucose ≥ 7.0 mmol/L, HbA1c ≥ 6.5%, or the use of any antidiabetic medication or self-reported diagnosis history of diabetes [22]. We used fasting glucose as a marker of glycemic control [23, 24]: good and poor glycemic control were defined as fasting glucose < 7.0 mmol/L and ≥ 7.0 mmol/L, respectively. The estimated glomerular filtration rate (eGFR) was calculated using the Chronic Kidney Disease Epidemiology Collaboration serum creatinine Eq.  (25). Diabetic nephropathy was defined as UACR ≥ 30 mg/mmol or eGFR < 60 mL/min/1.73m^2^ according to the Kidney Disease Improving Global Outcomes (KDIGO) [26–28]. Diabetic retinopathy was confirmed by ophthalmologists using the 45° four-field stereoscopic digital photography (Carl Zeiss Fundus Camera, Germany) based on the International Clinical Diabetic Retinopathy Disease Severity Scale [29].

### Arterial stiffness measurement and definition

Brachia-ankle pulse wave velocity (baPWV) reflects arterial stiffness status with high validity and repeatability and is a simple, noninvasive method for screening vascular damages in the general population [30]. At baseline, baPWV was measured using the Omron Colin BP-203RPE III device (Omron Health Care, Kyoto, Japan). Four pneumatic pressure cuffs were attached at the bilateral brachia and ankles and then connected to a plethysmographic sensor and oscillometric pressure sensor. The subjects were kept rest in supine position for at least 5 min in fasted condition. The maximum value of baPWV on the left and right sides was selected as the final value of arterial stiffness level. Arterial stiffness was divided into three groups according to baPWV values [31]: baPWV < 1400 cm/s indicates normal arterial stiffness, 1400 cm/s ≤ baPWV < 1800 cm/s indicates moderately elevated arterial stiffness and baPWV ≥ 1800 cm/s indicates severely elevated arterial stiffness.

### Outcome

The outcome of this present study was the occurrence of the following macrovascular complication: coronary artery disease, cerebrovascular disease, and peripheral arterial disease. Coronary heart disease and cerebrovascular disease were defined by self-reported diagnosis history, which were documented in the medical record system, and peripheral arterial disease was defined by an ankle-brachial index < 0.9. For those reporting history of cardiovascular events, the staff further confirmed the date and treatment experience to validate the accuracy of history reporting.

### Statistical analysis

Continuous variables with normal and skewed distributions were described as mean (SD) and median [IQR]. Differences were compared using Student's t-test or ANOVA for variables with normal distributions, and Mann–Whitney U test or Kruskal–Wallis test for skewed variables. Categorical variables were described as numbers (proportions) and compared using χ^2^ test. The follow-up time was calculated from baseline to the first occurrence of macrovascular complication or the end of study (December 31, 2019), whichever came first. The incidence rate of diabetic macrovascular complication was calculated by the number of incident cases divided by the total follow-up duration (per 1000 person-years).

We used two approaches to investigate the combined effect of arterial stiffness, glycemic control and hypertension status. Approach 1: the combined effect of arterial stiffness and glycemic control (or hypertension status) was evaluated separately; approach 2: we created two cumulative or weighted scores combining arterial stiffness, glycemic control and hypertension status. The detailed information was shown in Additional file 1: Additional methods.

Survival curves were used to present the cumulative hazard of diabetic macrovascular complications and compared by log-rank tests. Cox frailty models corrected for individual random intercept were used to compare the risk of macrovascular complication between groups. Hazard ratio (HR) with 95% confidence interval (CI) were calculated. We adjusted potential confounders in regression analyses: model 1 was adjusted for age and sex; model 2 was further adjusted for obesity, eGFR, LDL cholesterol, hypertension (if not stratified), glycemic control (if not stratified), current smoking, and physical activity. To visualize the dose–response relationship between baseline baPWV and incident macrovascular complication stratified by glycemic control and hypertension status, we carried out restricted cubic spline analysis using three knots at the 10th, 50th, and 90th percentiles.

Multiple sensitivity analyses were performed to test the robustness of the results. First, 62 of 1870 participants had missing data of BMI, and then the main analysis was repeated after completing the multiple imputation using Markov Chain Monte Carlo. Second, poor glycemic control was alternatively defined as fasting glucose ≥ 5.6 mmol/L and hypertension was alternatively defined as SBP ≥ 130 mmHg or DBP ≥ 80 mmHg or use of any antihypertensive medication or self-reported diagnosis history of hypertension. All statistical analyses were performed using R software (version 4.1.1), and a two-sided significance level of P value < 0.05 was considered statistically significant.

## Results

A total of 1 870 individuals with diabetes were included in this study, and 1227 (81.2%) were men. The mean age (SD) of the population was 59.90 (12.29) years. During a median follow-up of 4.0 years, 359 (19.2%) patients developed macrovascular complication. Table 1 shows the characteristics of individuals according to arterial stiffness status. We also analyzed the baseline characteristics stratified by the arterial stiffness level combined with glycemic control or hypertension status (Additional file 1: Tables S1, S2).

**Table 1:** Characteristics according to arterial stiffness level

	Arterial stiffness level			p value
	Normal	Moderate	Severe	
Participants, No	465	936	469	
Age (years)	51.50 (8.07)	58.42 (10.51)	71.17 (10.78)	< 0.001
Men, n (%)	373 (80.2)	773 (82.6)	374 (79.7)	0.346
BMI * (kg/m^2^)	26.79 (3.22)	27.63 (12.58)	26.14 (3.09)	0.015
Obesity * (n, %)	136 (30.4)	320 (35.3)	121 (26.7)	0.004
Physical activity (n, %)	219 (47.1)	413 (44.1)	210 (44.8)	0.570
Current smoking (n, %)	132 (28.4)	217 (23.2)	104 (22.2)	0.049
Current drinking (n, %)	295 (63.4)	513 (54.8)	247 (52.7)	0.002
Hypertension (n, %)	114 (24.5)	427 (45.6)	324 (69.1)	< 0.001
Antidiabetic (n, %)	65 (14.0)	200 (21.4)	145 (30.9)	< 0.001
Lipid lowering (n, %)	22 (4.7)	84 (9.0)	43 (9.2)	0.012
Antihypertensive (n, %)	48 (10.3)	181 (19.3)	136 (29.0)	< 0.001
Fasting glucose (mmol/L)	7.17 [5.80, 7.97]	7.09 [5.82, 7.97]	6.85 [5.71, 7.81]	0.089
Total cholesterol (mmol/L)	4.91 [4.24, 5.52]	4.87 [4.21, 5.55]	4.77 [4.08, 5.51]	0.177
Triglycerides (mmol/L)	1.59 [1.17, 2.43]	1.67 [1.13, 2.42]	1.39 [1.00, 1.94]	< 0.001
HDL cholesterol (mmol/L)	1.17 [1.01, 1.38]	1.18 [1.04, 1.37]	1.23 [1.08, 1.47]	< 0.001
LDL cholesterol (mmol/L)	3.08 [2.49, 3.64]	3.02 [2.46, 3.60]	2.91 [2.30, 3.47]	0.005
Uric acid (µmol/L)	340 [290, 406]	358 [306, 408]	353 [297, 404]	0.061
eGFR (mL/min/1.73 m^2^)	86.92 [76.72, 98.53]	83.41 [74.73, 93.99]	77.13 [69.46, 86.46]	< 0.001
BaPWV, cm/s	1311 [1247, 1359]	1564 [1480, 1669]	1975 [1879, 2145]	< 0.001
Macrovascular complication	37 (8.0)	166 (17.7)	156 (33.3)	< 0.001

Data are presented as mean (SD), median [IQR] or number (%)

Normal arterial stiffness refers to baPWV < 1400 cm/s, moderate arterial stiffness refers to 1400 ≤ baPWV < 1800 cm/s, severe arterial stiffness refers to baPWV ≥ 1800 cm/s. To convert fasting glucose to mg/dL, multiply by 18; triglycerides to mg/dL, multiply by 88.60; cholesterol to mg/dL, multiply by 38.66

BMI, body mass index; HDL, high-density lipoprotein; LDL, low-density lipoprotein; baPWV, brachial-ankle pulse wave velocity; eGFR, estimated glomerular filtration rate

^*^ Some individuals (n = 62) have missing data regarding the covariate

**Fig. 1:**
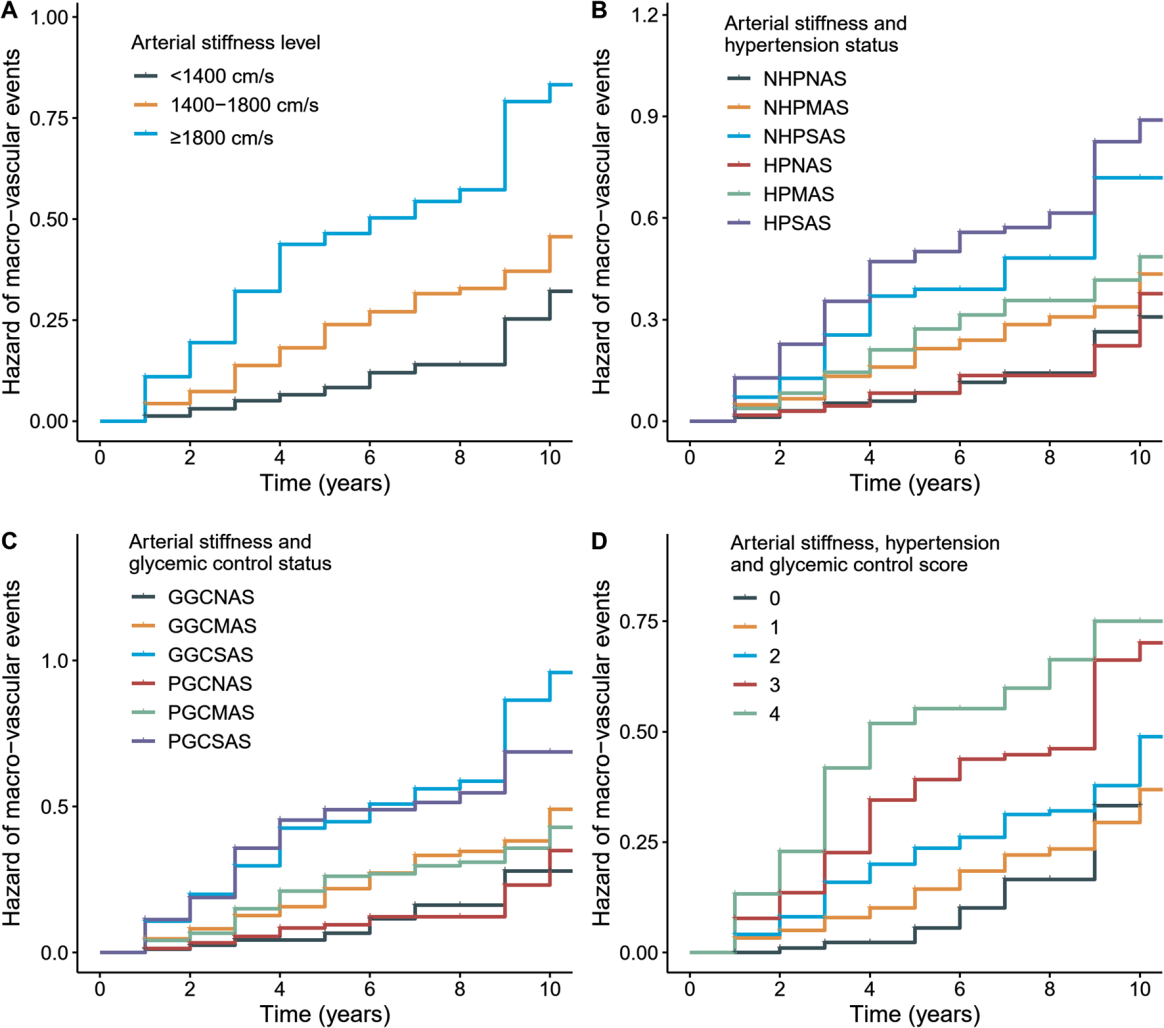
Cumulative risk curves of overall macrovascular complication according to arterial stiffness, glycemic control, and hypertension status. GGCNAS represents good glycemic control with normal arterial stiffness (baPWV < 1400 cm/s); GGCMAS, good glycemic control with moderately elevated arterial stiffness (1400 ≤ baPWV < 1800 cm/s); GGCSAS, good glycemic control with severely elevated arterial stiffness (baPWV ≥ 1800 cm/s); PGCNAS, poor glycemic control with normal arterial stiffness; PGCMAS, poor glycemic control with moderately elevated arterial stiffness; PGCSAS, poor glycemic control with severely elevated arterial stiffness. NHPNAS indicates no hypertension with normal arterial stiffness; NHPMAS, no hypertension with moderately elevated arterial stiffness; NHPSAS, no hypertension with severely elevated arterial stiffness; HPNAS, hypertension with normal arterial stiffness; HPMAS, hypertension with moderately elevated arterial stiffness; HPSAS, hypertension with severely elevated arterial stiffness. Score: moderately elevated arterial stiffness (1 score); severely elevated arterial stiffness (2 score); poor glycemic control (1 score); with hypertension (1 score)

Figure 1 shows the cumulative hazard of diabetic macrovascular complication. Then, we used Cox regression model to calculate the effect size of risk factors after adjusting the potential confounders. The fully adjusted HR (95% CI) for the association of baPWV with macrovascular complication was 1.29 (1.13–1.48) for per-SD increase of baPWV. Compared with people of baPWV < 1400 cm/s, those of 1400 ≤ baPWV ≤ 1800 cm/s and baPWV ≥ 1800 cm/s had higher risks of macrovascular complication, and the adjusted HR (95% CI) were 1.62 (1.09–2.42) and 2.27 (1.43–3.61), respectively (Additional file 1: Table S3).

Compared with the good glycemic control/normal arterial stiffness group, participants of poor glycemic control/severe arterial stiffness had the highest risk of macrovascular complication (HR: 2.73; 95% CI: 1.42–5.25). The adjusted HR for good glycemic control/severe arterial stiffness group was 2.24 (95% CI: 1.17–4.30); and 1.98 (95% CI: 1.08–3.61) for poor glycemic control/moderate arterial stiffness group. No significant association was found for the poor glycemic control/normal arterial stiffness and good glycemic control/moderate arterial stiffness groups (Table 2). Similarly, compared with the non-hypertension/normal arterial stiffness group, participants of hypertension/severe arterial stiffness had the highest risk of macrovascular complication (HR: 2.69; 95% CI: 1.61–4.50). The adjusted HRs for hypertension/moderate arterial stiffness and non-hypertension/severe arterial stiffness groups were 1.93 (95% CI: 1.19–3.12) and 1.92 (95% CI: 1.08–3.43), respectively. No significant association was found for the hypertension/normal arterial stiffness and non-hypertension/moderate arterial stiffness groups (Table 3). Results were similar when adjusting fasting glucose and SBP as continuous variables, except the poor glycemic control/moderate arterial stiffness group became nonsignificant (P = 0.063) as shown in Additional file 1: Table S4. Consistent results were observed after imputation for missing data (Additional file 1: Figure S2), using 5.6 mmol/L as cutoff points of glycemic control (Additional file 1: Table S5), and 130/80 mm Hg as the diagnostic threshold of hypertension (Additional file 1: Table S6).

**Table 2:** Association of combined arterial stiffness and glycemic control status with macrovascular complication

	Cases/total (%)	Incidence rate (per 1000 person years)	Hazard ratio (95% CI)	P value
*Model 1*				
Good glycemic control and normal arterial stiffness	15/183 (8.2)	19.3	Ref.	
Good glycemic control and moderate arterial stiffness	82/415 (19.8)	43.6	1.63 (0.91–2.92)	0.103
Good glycemic control and severe arterial stiffness	91/246 (37.0)	88.6	2.34 (1.26–4.35)	0.007
Poor glycemic control and normal arterial stiffness	22/282 (7.8)	21.1	1.14 (0.58–2.26)	0.698
Poor glycemic control and moderate arterial stiffness	84/521 (16.1)	42.6	1.87 (1.05–3.34)	0.033
Poor glycemic control and severe arterial stiffness	65/223 (29.1)	84.3	2.65 (1.42–4.95)	0.002
*Model 2*				
Good glycemic control and moderate arterial stiffness			1.57 (0.85–2.89)	0.147
Good glycemic control and severe arterial stiffness			2.24 (1.17–4.30)	0.016
Poor glycemic control and normal arterial stiffness			1.19 (0.59–2.41)	0.632
Poor glycemic control and moderate arterial stiffness			1.98 (1.08–3.61)	0.027
Poor glycemic control and severe arterial stiffness			2.73 (1.42–5.25)	0.003

Normal arterial stiffness (baPWV < 1400 cm/s); moderate arterial stiffness (1400 ≤ baPWV < 1800 cm/s); severe arterial stiffness (baPWV ≥ 1800 cm/s). Good and poor glycemic control were defined as fasting glucose < 7.0 mmol/L and ≥ 7.0 mmol/L

Model 1: adjusted for age (continuous) and sex; model 2: adjusted for age (continuous), sex, obesity (yes/no), eGFR (continuous), LDL cholesterol (continuous), hypertension (yes/no), current smoking (yes/no) and physical activity (yes/no)

baPWV, brachial-ankle pulse wave velocity; LDL, low-density lipoprotein; eGFR, estimated glomerular filtration rate

**Table 3:** Association of combined arterial stiffness and hypertension status with macrovascular complication

	Cases/total (%)	Incidence rate (per 1000 person years)	Hazard Ratio (95% CI)	P value
*Model 1*				
Non-hypertension and normal arterial stiffness	27/351 (7.7)	20.0	Ref.	
Non-hypertension and moderate arterial stiffness	87/509 (17.1)	39.9	1.50 (0.94–2.40)	0.088
Non-hypertension and severe arterial stiffness	41/145 (28.3)	70.4	1.84 (1.04–3.27)	0.037
Hypertension and normal arterial stiffness	10/114 (8.8)	21.2	0.98 (0.46–2.09)	0.955
Hypertension and moderate arterial stiffness	79/427 (18.5)	47.2	1.81 (1.12–2.90)	0.015
Hypertension and severe arterial stiffness	115/324 (35.5)	94.6	2.64 (1.59–4.39)	< 0.001
*Model 2*				
Non-hypertension and moderate arterial stiffness			1.42 (0.88–2.29)	0.148
Non-hypertension and severe arterial stiffness			1.92 (1.08–3.43)	0.027
Hypertension and normal arterial stiffness			0.90 (0.41–1.97)	0.784
Hypertension and moderate arterial stiffness			1.93 (1.19–3.12)	0.008
Hypertension and severe arterial stiffness			2.69 (1.61–4.50)	< 0.001

Normal arterial stiffness (baPWV < 1400 cm/s); moderate arterial stiffness (1400 ≤ baPWV < 1800 cm/s); severe arterial stiffness (baPWV ≥ 1800 cm/s). Hypertension was defined as systolic blood pressure ≥ 140 mmHg or diastolic blood pressure ≥ 90 mmHg or use of any antihypertensive medication or self-reported diagnosis history of hypertension

Model 1: adjusted for age (continuous) and sex; model 2: adjusted for age (continuous), sex, obesity (yes/no), eGFR (continuous), LDL cholesterol (continuous), glycemic control (good/poor), current smoking (yes/no) and physical activity (yes/no)

baPWV, brachial-ankle pulse wave velocity; LDL, low-density lipoprotein; eGFR, estimated glomerular filtration rate

**Fig. 2:**
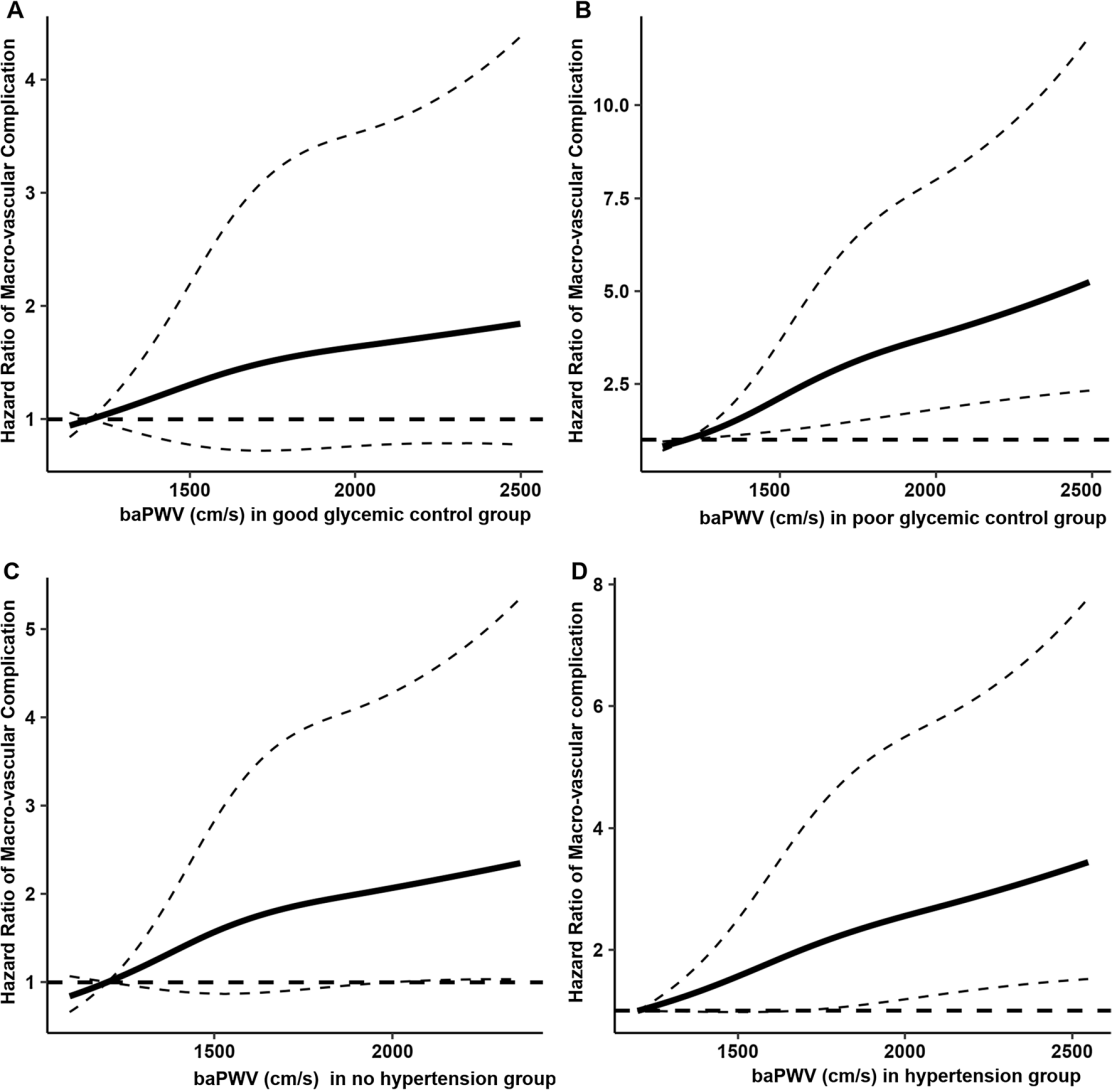
Dose–response relationships of baseline arterial stiffness level with incident macrovascular complication stratified by glycemic control and hypertension status. Restricted cubic spline regression model was conducted using 3 knots at the 10th, 50th, and 90th percentiles, using 1200 cm/s as reference. Black solid line represents the hazard ratio and the dashed line represents the 95% CI. The y-axis ranges of **A**–**D** are different. Analyses were adjusted for age (continuous), sex, obesity (yes/no), eGFR (continuous), LDL cholesterol (continuous), hypertension (yes/no, if not stratified), glycemic control (good/poor, if not stratified), current smoking (yes/no) and physical activity (yes/no). baPWV, brachial-ankle pulse wave velocity; LDL, low-density lipoprotein; eGFR, estimated glomerular filtration rate

Figure 2 presents the restricted cubic spline curves stratified by glycemic control and hypertension status, indicating a significant dose–response relationship between baseline baPWV and macrovascular complication in poor glycemic control and hypertension groups, not in good glycemic control and non-hypertension groups. Additional file 1: Table S7 summarizes the association of per-SD increase of baPWV with macrovascular complication stratified by glycemic control and hypertension status.

**Fig. 3:**
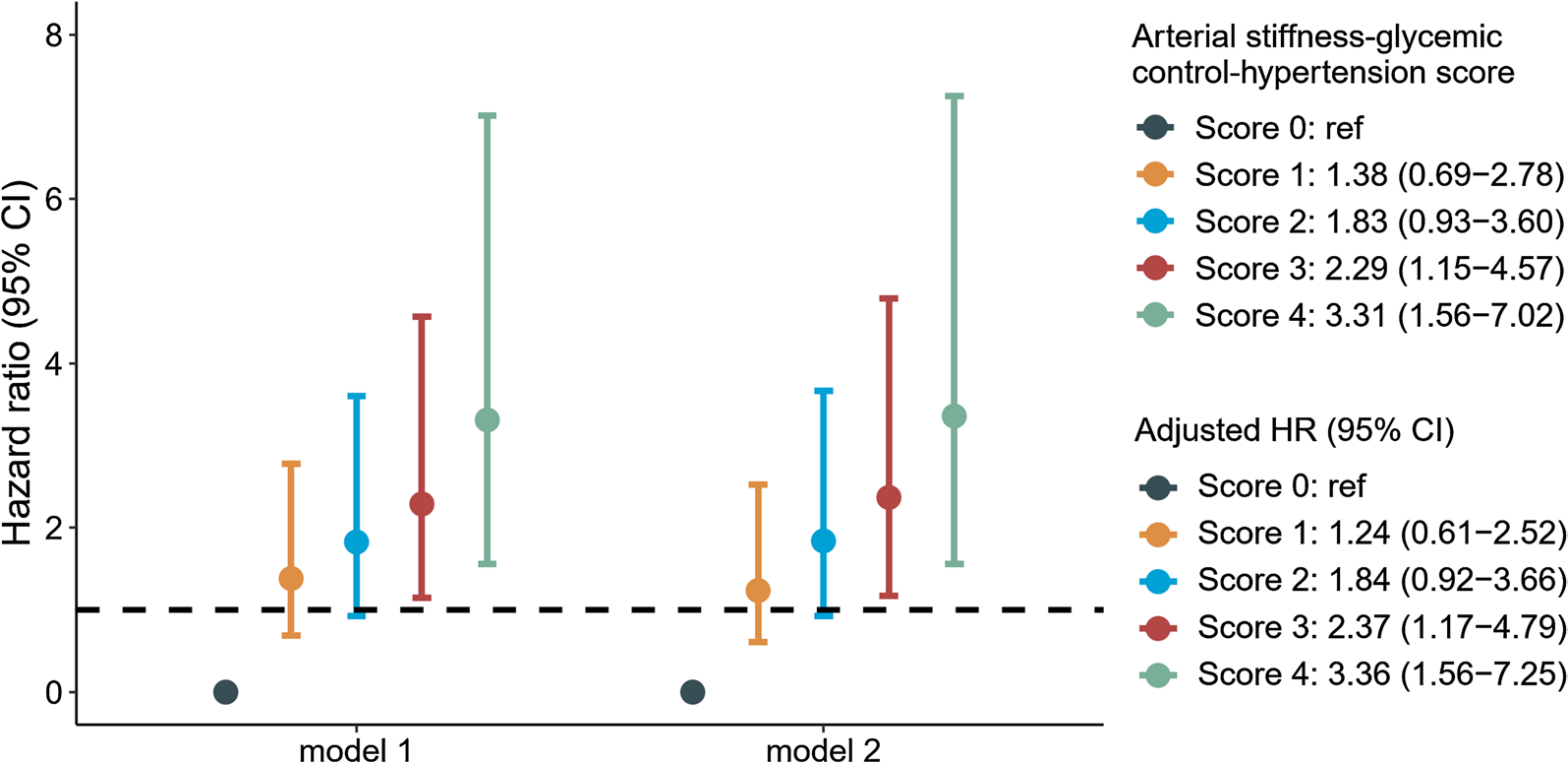
Association of cumulative score by arterial stiffness, glycemic control and hypertension with the development of macrovascular complication. Score represents the cumulative risk of moderate arterial stiffness (1 score), severe arterial stiffness (2 score), poor glycemic control (1 score) and hypertension (1 score). Analyses were adjusted for age (continuous), sex, obesity (yes/no), eGFR (continuous), LDL cholesterol (continuous), current smoking (yes/no) and physical activity (yes/no)

Figure 3 shows the association of the cumulative risk score of arterial stiffness, glycemic control, and hypertension with macrovascular complication. Individuals in the highest composite risk score group had a significantly highest risk of macrovascular complication (adjusted HR: 3.36; 95% CI: 1.56–7.25) and the adjusted HR for the group of score 3 was 2.37 (95% CI: 1.17–4.79) compared with those of score 0. The composition of arterial stiffness, glycemic control and hypertension status according to the cumulative risk score is shown in Additional file 1: Table S8. On the other hand, one-unit increase of the weighted score was associated with a 21.4% (95% CI: 12.4%-43.1%) increased risk of macrovascular complication (Additional file 1: Table S9). The area under the curve (AUC) values were 0.854 and 0.893 for the cumulative score and weighted score, respectively.

## Discussion

This current cohort study examined the macrovascular complication risk among diabetic population based on the combined evaluation of arterial stiffness, glycemic control and hypertension status. We found that individuals with elevated arterial stiffness were at a higher risk of macrovascular complication. Among patients of normal blood pressure or good glycemic control, the risk of macrovascular complication was only significant for the severe arterial stiffness. Whereas, in patients with hypertension or poor glycemic control, the effect became significant from the moderate arterial stiffness. In addition, we established a cumulative and weighted risk score by arterial stiffness, glycemic control and hypertension status for the risk stratification of diabetic macrovascular complication. The results were consistent in multiple sensitivity analyses.

Separate associations of arterial stiffness, glycemic control and hypertension with diabetic macrovascular complication have been reported. Previous studies showed a positive association between arterial stiffness and cardiovascular diseases in patients with diabetes [32–35]. The Diabetes Control and Complications Trial (DCCT) [36], Action in Diabetes and Vascular Disease: Preterax and Diamicron Modified Release Controlled Evaluation (ADVANCE) [37], and the UK Prospective Diabetes Study (UKPDS) trial [38] have shown that good glycemic control reduced macrovascular complication onset. Intensive glycemic control could modestly reduce major macrovascular events in the short-to-medium term. On contrary, some studies failed to demonstrate a significant association [39–41]. Our study showed that glycemic control status was not significantly associated with macrovascular complication, but it could modulate the effect of arterial stiffness. On the other hand, the UKPDS showed that a reduction in SBP decreased the risk of myocardial infarction, stroke, and peripheral artery disease [42]. Intensive blood pressure control decreased the risk of cardiovascular outcomes in diabetic population [43, 44]. On this basis, this study proposed that combined evaluation of arterial stiffness, glycemic control and hypertension status could improve the risk stratification of macrovascular complication onset in diabetic population.

A previous study investigated the significance of combined evaluation of arterial stiffness and hypertension status on diabetes onset [18]. The Framingham offspring cohort study found the relative contributions of arterial stiffness and hypertension to cardiovascular diseases [45]. However, the combined association of arterial stiffness, glycemic control and hypertension status with macrovascular complication among diabetic patients remained unknown. Our study evaluated the joint association of arterial stiffness, glycemic control and hypertension status with the incidence of macrovascular complication. Moreover, we established two composite risk scores to stratify the risk of macrovascular complication, which provided a comprehensive tool for the risk stratification and management of macrovascular complication in diabetic population.

The mutual associations between arterial stiffness, glycemic control and hypertension have been extensively documented. A Japanese study showed that an increase of arterial stiffness appeared to be associated with a longitudinal elevation of blood pressure to the hypertensive range [46]. The Rio de Janeiro diabetes cohort study found that better glycemic control with blood pressure reduction were of great importance to attenuate arterial stiffness progression in diabetic patients [15]. Our study found that in patients of non-hypertension or good glycemic control, the effect was statistically significant only for severe arterial stiffness. Our results indicated that the combined action of arterial stiffness, blood pressure and glycose level on macrovascular complication needs to be considered and managed accordingly to most efficiently reduce CVD risk. In addition, people of diabetes are at a higher risk of heart failure [47, 48]. The multifactorial risk prevention and control of arterial stiffness, glycemic and blood pressure could potentially reduce the incidence of heart failure, which needs further research.

The exact mechanisms linking arterial stiffness and macrovascular complication remain unclear and there are some potential explanations. First, arterial stiffness could lead to increased arterial pulse pressure and pulsatile shear, resulting in endothelial dysfunction and metabolic dysregulation [49]. Second, arterial stiffness could lead to capillary diastolic dysfunction or loss which results in tissue perfusion decline [31]. The effect of glycemic control on arterial stiffness may be mediated by advanced glycation end product (AGE) formation. Hyperglycemia may increase the reaction between glucose and proteins, promoting the cross-linking of collagen, elastin and other molecules, known as AGE, which could produce collagen deposits, tissue inflammation, and fibrosis within the vessel wall [50]. And the lower production of AGE associated with good glycemic control may delay the progression of arterial stiffness [51].

Our study was based on a large cohort of diabetic population and reported the longitudinal association of combined arterial stiffness, glycemic control and hypertension status with macrovascular complication. Data obtained from the central medical system and standardized questionnaire allowed us to adjust for the potential confounding factors. However, several limitations should be acknowledged. First, only part (56%) of the population had measurements of HbA1c. Fasting blood glucose was used as a measure of glycemic control. Second, we used baPWV as the index of arterial stiffness instead of carotid-femoral pulse wave velocity (cfPWV). We can not compare the difference between these two indices. Nevertheless, studies have confirmed that baPWV correlates closely with cfPWV and has been recommended for assessing arterial stiffness by American Heart Association (AHA). Of note, there are novel techniques to estimate the arterial stiffness in addition to tonometry-derived measures, such as Doppler-derived aortic arch PWV (aa-PWV), which need investigation in further research among diabetic population [52]. Third, arterial stiffness, glycemic control and blood pressure were determined at baseline, and further studies are needed to underline the dynamic changes of glycemic level and blood pressure during follow-up. Fourth, the diseases history of cardiovascular diseases was self-reported, which could cause a recall-bias. And there could exist other potential confounding factors due to the observational study design.

## Conclusion

In summary, this longitudinal cohort study indicated that the combined evaluation of arterial stiffness, glycemic control and hypertension status could improve the risk stratification of macrovascular complication among diabetic population, which provided novel insights into the preventive strategies against macrovascular complication from the perspective of combined intervention of arterial function and glucose metabolism.

## Supplementary Information

**Additional file 1. **Additional Methods.** Table S1. **Baseline characteristics of participants according to different arterial stiffness and glycemic control status. **Table S2.** Baseline characteristics of participants according to different arterial stiffness and hypertension status. **Table S3.** Association of separate arterial stiffness, glycemic control and hypertension status with the development of macrovascular complication. **Table S4.** Association of arterial stiffness and glycemic control/hypertension status with macrovascular complications adjusting blood pressure and fasting glucose as continuous variables. **Table S5.** Association of arterial stiffness and alternatively defined glycemic control status with macrovascular complications. **Table S6.** Association of arterial stiffness and alternatively defined hypertension status with macrovascular complications. **Table S7.** Association of per-SD increase of baPWV with diabetic macrovascular complication stratified by glycemic control and hypertension. **Table S8.** The composition of arterial stiffness, glycemic control and hypertension in each scored population. **Table S9.** Association of weighted score with diabetic macrovascular complications. **Figure S1.** Flowchart of this current study. **Figure S2.** Association of arterial stiffness, glycemic control and hypertension status with development of macrovascular complication in imputed data.

## Data Availability

The datasets used and/or analysed during the current study are available from the corresponding author (Dr. Xiuhua Guo) on reasonable request.

## Abbreviations

BHMC: Beijing Health Management Cohort
baPWV: Brachia-ankle pulse wave velocity
SBP: Systolic blood pressure
DBP: Diastolic blood pressure
BMI: Body mass index
HDL: High-density lipoprotein
LDL: Low-density lipoprotein
UACR: Urinary albumin/creatinine ratio
eGFR: Estimated glomerular filtration rate
cfPWV: Carotid-femoral pulse wave velocity
AGE: Advanced glycation end product
