# Supplementary material for: Combined evaluation of arterial stiffness, glycemic control and hypertension for macrovascular complications in type 2 diabetes
Source: Cardiovasc Diabetol. 2022 Nov 28;21:262. doi: 10.1186/s12933-022-01696-1 (PMC9706858; doi:10.1186/s12933-022-01696-1)
Supplement: Supplementary file 1 — Additional file 1. Additional Methods. Table S1. Baseline characteristics of participants according to different arterial stiffness and glycemic control status. Table S2. Baseline characteristics of participants according to different arterial stiffness and hypertension status. Table S3. Association of separate arterial stiffness, glycemic control and hypertension status with the development of macrovascular complication. Table S4. Association of arterial stiffness and glycemic control/hypertension status with macrovascular complications adjusting blood pressure and fasting glucose as continuous variables. Table S5. Association of arterial stiffness and alternatively defined glycemic control status with macrovascular complications. Table S6. Association of arterial stiffness and alternatively defined hypertension status with macrovascular complications. Table S7. Association of per-SD increase of baPWV with diabetic macrovascular complication stratified by glycemic control and hypertension. Table S8. The composition of arterial stiffness, glycemic control and hypertension in each scored population. Table S9. Association of weighted score with diabetic macrovascular complications. Figure S1. Flowchart of this current study. Figure S2. Association of arterial stiffness, glycemic control and hypertension status with development of macrovascular complication in imputed data. [file 12933_2022_1696_MOESM1_ESM.docx]

**Additional Material**

**Combined evaluation of arterial stiffness, glycemic control and hypertension for macrovascular complications in type 2 diabetes**

Zhiyuan Wu, et al.

1. **Additional Methods**
2. **Additional Tables**

**Table S1:** Baseline characteristics of participants according to different arterial stiffness and glycemic control status.

**Table S2:** Baseline characteristics of participants according to different arterial stiffness and hypertension status.

**Table S3:** Association of separate arterial stiffness, glycemic control and hypertension status with the development of macrovascular complication.

**Table S4**: Association of arterial stiffness and glycemic control/hypertension status with macrovascular complications adjusting blood pressure and fasting glucose as continuous variables.

**Table S5**: Association of arterial stiffness and alternatively defined glycemic control status with macrovascular complications.

**Table S6**: Association of arterial stiffness and alternatively defined hypertension status with macrovascular complications.

**Table S7**: Association of per-SD increase of baPWV with diabetic macrovascular complication stratified by glycemic control and hypertension.

**Table S8**: The composition of arterial stiffness, glycemic control and hypertension in each scored population.

**Table S9**: Association of weighted score with diabetic macrovascular complications.

1. **Additional Figures**

**Figure S1:** Flowchart of this current study.

**Figure S2:** Association of arterial stiffness, glycemic control and hypertension status with development of macrovascular complication in imputed data.

**Additional Methods**

Participants were then divided into six groups considering arterial stiffness and glycemic control status: (1) good glycemic control with ideal arterial stiffness (control group); (2) good glycemic control with moderately elevated arterial stiffness; (3) good glycemic control with severely elevated arterial stiffness; (4) poor glycemic control with ideal arterial stiffness; (5) poor glycemic control with moderately elevated arterial stiffness; (6) poor glycemic control with severely elevated arterial stiffness, or hypertension status: (1) no hypertension with ideal arterial stiffness (control group); (2) no hypertension with moderately elevated arterial stiffness; (3) no hypertension with severely elevated arterial stiffness; (4) hypertension with normal arterial stiffness; (5) hypertension with moderately elevated arterial stiffness; (6) hypertension with severely elevated arterial stiffness.

Then, we developed two scores combining arterial stiffness, glycemic control and hypertension status. The first score was a simple cumulative risk score (0-4 points) summarizing the situations of poor glycemic control (1 point), with hypertension (1 point) and arterial stiffness severity (1 point: moderately elevated arterial stiffness; 2 point: severely elevated arterial stiffness); the second one was a weighted score considering the effect size of arterial stiffness (2 point: moderately elevated arterial stiffness; 3 points: severely elevated arterial stiffness), poor glycemic control (1 point) and hypertension (1 point) on diabetic macrovascular diseases.

**Additional Tables**

**Table S1:** Baseline characteristics of participants according to different arterial stiffness and glycemic control status.

|  | NDMNAS | NDMMAS | NDMSAS | DMNAS | DMMAS | DMSAS | P value |
| --- | --- | --- | --- | --- | --- | --- | --- |
| Participants, No. | 183 | 415 | 246 | 282 | 521 | 223 |  |
| Age (years) | 52.27 (8.51) | 60.96 (10.40) | 73.24 (9.11) | 51.00 (7.75) | 56.40 (10.17) | 68.89 (11.97) | <0.001 |
| Men, n (%) | 133 (72.7) | 324 (78.1) | 187 (76.0) | 240 (85.1) | 449 (86.2) | 187 (83.9) | <0.001 |
| BMI* (kg/m2) | 25.92 (2.96) | 26.49 (3.10) | 25.53 (2.93) | 27.38 (3.27) | 28.55 (16.61) | 26.82 (3.14) | <0.001 |
| Obesity* (n, %) | 42 (23.5) | 123 (30.4) | 50 (20.8) | 95 (35.4) | 197 (39.2) | 71 (33.2) | <0.001 |
| Physical activity (n, %) | 94 (51.4) | 185 (44.6) | 114 (46.3) | 125 (44.3) | 228 (43.8) | 96 (43.0) | 0.561 |
| Current smoking (n, %) | 42 (23.0) | 84 ( 20.2) | 52 (21.1) | 90 (31.9) | 133 (25.5) | 52 (23.3) | 0.012 |
| Current drinking (n, %) | 106 (57.9) | 213 (51.3) | 130 (52.8) | 189 (67.0) | 300 (57.6) | 117 (52.5) | 0.001 |
| Hypertension (n, %) | 61 (33.3) | 219 (52.8) | 187 (76.0) | 53 (18.8) | 208 (39.9) | 137 (61.4) | <0.001 |
| Antidiabetic (n, %) | 53 (29.0) | 174 (41.9) | 126 (51.2) | 12 (4.3) | 26 (5.0) | 19 (8.5) | <0.001 |
| Lipid lowering (n, %) | 18 (9.8) | 67 (16.1) | 35 (14.2) | 4 (1.4) | 17 (3.3) | 8 (3.6) | 0.012 |
| Antihypertensive (n, %) | 33 (18.0) | 120 (28.9) | 102 (41.5) | 15 (5.3) | 61 (11.7) | 34 (15.2) | <0.001 |
| Fasting glucose (mmol/L) | 5.54 [5.15, 6.06] | 5.69 [5.27, 6.26] | 5.74 [5.26, 6.26] | 7.74 [7.25, 8.75] | 7.81 [7.30, 9.01] | 7.88 [7.39, 9.14] | <0.001 |
| Total cholesterol (mmol/L) | 4.87 [4.12, 5.52] | 4.74 [4.11, 5.47] | 4.86 [4.10, 5.47] | 4.94 [4.29, 5.54] | 4.99 [4.24, 5.64] | 4.68 [4.04, 5.51] | 0.038 |
| Triglycerides (mmol/L) | 1.34 [1.04, 1.93] | 1.45 [1.02, 2.12] | 1.27 [0.97, 1.73] | 1.89 [1.33, 2.70] | 1.85 [1.25, 2.69] | 1.63 [1.08, 2.22] | <0.001 |
| HDL cholesterol (mmol/L) | 1.22 [1.04, 1.46] | 1.22 [1.08, 1.41] | 1.26 [1.09, 1.51] | 1.14 [1.00, 1.33] | 1.14 [1.02, 1.33] | 1.17 [1.06, 1.40] | <0.001 |
| LDL cholesterol (mmol/L) | 3.11 [2.50, 3.57] | 2.92 [2.39, 3.57] | 2.92 [2.28, 3.50] | 3.05 [2.50, 3.68] | 3.09 [2.51, 3.62] | 2.90 [2.34, 3.47] | 0.011 |
| Uric acid (µmol/L) | 340.00 [287.80, 403.90] | 360.30 [307.00, 414.00] | 363.00 [306.55, 411.15] | 340.10 [290.75, 406.45] | 355.50 [303.00, 403.00] | 336.00 [288.95, 390.50] | 0.009 |
| eGFR (mL/min/1.73 m2) | 83.58 [75.15, 96.03] | 80.89 [72.26, 91.49] | 75.50 [68.77, 84.28] | 87.77 [77.58, 100.58] | 85.63 [76.55, 96.85] | 79.99 [70.07, 89.29] | <0.001 |
| BaPWV, cm/s | 1305.00 [1240.50, 1353.00] | 1576.00 [1483.50, 1665.50] | 1969.50 [1879.00, 2103.75] | 1317.50 [1258.00, 1361.75] | 1554.00 [1474.00, 1673.00] | 1990.00 [1877.50, 2167.00] | <0.001 |
| Macrovascular complications | 15 (8.2) | 82 (19.8) | 91 (37.0) | 22 (7.8) | 84 (16.1) | 65 (29.1) | <0.001 |

Data are presented as mean (SD), median [IQR] or number (%).

To convert fasting glucose to mg/dL, multiply by 18; triglycerides to mg/dL, multiply by 88.60; cholesterol to mg/dL, multiply by 38.66.

Abbreviations: BMI, body mass index; HDL, high-density lipoprotein; LDL, low-density lipoprotein; BaPWV, brachial-ankle pulse wave velocity. GGCNAS represents good glycemic control with normal arterial stiffness (baPWV <1400 cm/s); GGCMAS, good glycemic control with moderately elevated arterial stiffness (1400≤ baPWV <1800 cm/s); GGCSAS, good glycemic control with severely elevated arterial stiffness (baPWV ≥1800 cm/s); PGCNAS, poor glycemic control with normal arterial stiffness; PGCMAS, poor glycemic control with moderately elevated arterial stiffness; PGCSAS, poor glycemic control with severely elevated arterial stiffness.

*Some individuals have missing data regarding these covariates.

**Table S2:** Baseline characteristics of participants according to different arterial stiffness and hypertension status.

|  | NHPNAS | NHPMAS | NHPSAS | HPNAS | HPMAS | HPSAS | P value |
| --- | --- | --- | --- | --- | --- | --- | --- |
| Participants, No. | 351 | 509 | 145 | 114 | 427 | 324 |  |
| Age (years) | 51.18 (7.76) | 58.63 (9.96) | 70.97 (10.48) | 52.50 (8.93) | 58.17 (11.14) | 71.27 (10.93) | <0.001 |
| Men, n (%) | 279 (79.5) | 421 (82.7) | 123 (84.8) | 94 (82.5) | 352 (82.4) | 251 (77.5) | 0.288 |
| BMI* (kg/m2) | 26.72 (3.25) | 27.68 (16.80) | 25.99 (2.72) | 27.01 (3.15) | 27.58 (3.28) | 26.21 (3.25) | 0.125 |
| Obesity* (n, %) | 96 (28.7) | 143 (29.0) | 35 (25.2) | 41 (36.3) | 177 (42.8) | 86 (27.3) | <0.001 |
| Physical activity (n, %) | 167 (47.6) | 225 (44.2) | 68 (46.9) | 52 (45.6) | 188 (44.0) | 142 (43.8) | 0.896 |
| Current smoking (n, %) | 453 (24.2) | 103 (29.3) | 117 (23.0) | 34 (23.4) | 29 (25.4) | 100 (23.4) | 0.225 |
| Current drinking (n, %) | 228 (65.0) | 274 (53.8) | 79 (54.5) | 67 (58.8) | 239 (56.0) | 168 (51.9) | 0.01 |
| Hypertension (n, %) | 0 (0.0) | 0 (0.0) | 0 (0.0) | 114 (100.0) | 427 (100.0) | 324 (100.0) | <0.001 |
| Antidiabetic (n, %) | 36 (10.3) | 81 (15.9) | 28 (19.3) | 29 (25.4) | 119 (27.9) | 117 (36.1) | <0.001 |
| Lipid lowering (n, %) | 6 (1.7) | 24 (4.7) | 5 (3.4) | 16 (14.0) | 60 (14.1) | 38 (11.7) | 0.012 |
| Antihypertensive (n, %) | 0 (0.0) | 0 (0.0) | 0 (0.0) | 48 (42.1) | 181 (42.4) | 136 (42.0) | <0.001 |
| Fasting glucose (mmol/L) | 7.23 [6.11, 8.14] | 7.21 [6.16, 8.01] | 7.30 [6.17, 8.09] | 6.62 [5.44, 7.62] | 6.94 [5.64, 7.92] | 6.50 [5.60, 7.61] | <0.001 |
| Total cholesterol (mmol/L) | 4.91 [4.26, 5.55] | 4.84 [4.12, 5.55] | 4.59 [4.01, 5.38] | 4.86 [4.18, 5.48] | 4.90 [4.30, 5.55] | 4.82 [4.12, 5.52] | 0.215 |
| Triglycerides (mmol/L) | 1.68 [1.22, 2.42] | 1.62 [1.08, 2.41] | 1.39 [0.99, 1.94] | 1.46 [1.13, 2.57] | 1.71 [1.16, 2.45] | 1.40 [1.02, 1.94] | <0.001 |
| HDL cholesterol (mmol/L) | 1.18 [1.02, 1.39] | 1.17 [1.03, 1.36] | 1.23 [1.07, 1.40] | 1.14 [0.99, 1.33] | 1.18 [1.05, 1.38] | 1.23 [1.08, 1.50] | 0.002 |
| LDL cholesterol (mmol/L) | 3.10 [2.50, 3.65] | 3.00 [2.43, 3.57] | 2.89 [2.31, 3.42] | 3.02 [2.51, 3.62] | 3.04 [2.47, 3.64] | 2.91 [2.30, 3.54] | 0.033 |
| Uric acid (µmol/L) | 338.30 [287.75, 407.85] | 353.50 [307.80, 405.00] | 357.00 [299.80, 418.00] | 345.45 [296.25, 399.25] | 363.00 [302.00, 412.50] | 351.25 [295.38, 397.45] | 0.088 |
| eGFR (mL/min/1.73 m2) | 86.90 [76.30, 97.88] | 83.81 [75.39, 94.04] | 77.36 [69.10, 84.60] | 87.48 [77.31, 101.53] | 82.64 [73.32, 93.95] | 76.65 [69.97, 87.40] | <0.001 |
| BaPWV, cm/s | 1304.00 [1240.00, 1353.50] | 1540.00 [1467.00, 1658.00] | 1960.00 [1883.00, 2136.00] | 1336.00 [1281.50, 1372.00] | 1583.00 [1495.00, 1676.00] | 1982.50 [1877.50, 2164.00] | <0.001 |
| Macrovascular complications | 27 (7.7) | 87 (17.1) | 41 (28.3) | 10 (8.8) | 79 (18.5) | 115 (35.5) | <0.001 |

Data are presented as mean (SD), median [IQR] or number (%).

To convert fasting glucose to mg/dL, multiply by 18; triglycerides to mg/dL, multiply by 88.60; cholesterol to mg/dL, multiply by 38.66.

Abbreviations: BMI, body mass index; HDL, high-density lipoprotein; LDL, low-density lipoprotein; BaPWV, brachial-ankle pulse wave velocity. NHPNAS indicates no hypertension with normal arterial stiffness (baPWV <1400 cm/s); NHPMAS, no hypertension with moderately elevated arterial stiffness (1400≤ baPWV <1800 cm/s); NHPSAS, no hypertension with severely elevated arterial stiffness (baPWV ≥1800 cm/s); HPNAS, hypertension with normal arterial stiffness; HPMAS, hypertension with moderately elevated arterial stiffness; HPSAS, hypertension with severely elevated arterial stiffness.

*Some individuals have missing data regarding these covariates.

**Table S3:** Association of separate arterial stiffness, glycemic control and hypertension status with the development of macrovascular complication.

|  | Hazard Ratio (95% CI) | | | |
| --- | --- | --- | --- | --- |
|  | Model 1 | P value | Model 2 | P value |
| baPWV, per-SD | 1.31(1.15-1.50) | <0.001 | 1.29(1.13-1.48) | <0.001 |
| baPWV <1400 cm/s | Ref |  | Ref |  |
| 1400≤ baPWV <1800 cm/s ^a^ | 1.63(1.11-2.40) | 0.013 | 1.62(1.09-2.42) | 0.018 |
| baPWV ≥1800 cm/s ^a^ | 2.35(1.51-3.66) | <0.001 | 2.27(1.43-3.61) | <0.001 |
| P for trend | 1.51(1.23-1.86) | <0.001 | 1.50(1.20-1.88) | <0.001 |
| fasting glucose, per-SD | 1.062(0.949-1.188) | 0.297 | 1.059(0.932-1.203) | 0.379 |
| good glycemic control | ref |  | ref |  |
| poor glycemic control | 1.117(0.896-1.392) | 0.327 | 1.197(0.939-1.526) | 0.147 |
| SBP, per-SD |  |  |  |  |
| non-hypertension | ref |  | ref |  |
| hypertension ^a^ | 1.325(1.07-1.641) | 0.010 | 1.318(1.032-1.684) | 0.027 |

Normal arterial stiffness (baPWV <1400 cm/s); moderate arterial stiffness (1400≤ baPWV <1800 cm/s); severe arterial stiffness (baPWV ≥1800 cm/s). Good and poor glycemic control were defined as fasting glucose <7.0 mmol/L and ≥7.0 mmol/L. Poor glycemic control is defined as fasting glucose ≥ 7.0 mmol/L. Hypertension was defined as systolic blood pressure ≥140 mmHg or diastolic blood pressure ≥90 mmHg, the use of any antihypertensive medication, or self-reported diagnosis history of hypertension.

Model 1: adjusted for age (continuous) and sex; model 2: adjusted for age (continuous), sex, obesity (yes/no), eGFR (continuous), LDL cholesterol (continuous), hypertension (yes/no, if not stratified), glycemic control (good/poor, if not stratified), current smoking (yes/no), physical activity (yes/no), baPWV level (if not stratified).

Abbreviations: baPWV, brachial-ankle pulse wave velocity; SBP, systolic blood pressure; LDL, low-density lipoprotein; eGFR, estimated glomerular filtration rate.

^a^ indicates P value <0.05.

**Table S4:** Association of arterial stiffness and glycemic control/hypertension status with macrovascular complications adjusting blood pressure and fasting glucose as continuous variables.

|  | Model 1 | | Model 2 | |
| --- | --- | --- | --- | --- |
|  | HR (95% CI) | P value | HR (95% CI) | P value |
| Good glycemic control and normal arterial stiffness | ref |  | ref |  |
| Good glycemic control and moderate arterial stiffness | 1.626(0.907-2.915) | 0.103 | 1.454(0.751-2.814) | 0.267 |
| Good glycemic control and severe arterial stiffness ^a^ | 2.341(1.26-4.348) | 0.007 | 2.371(1.149-4.894) | 0.020 |
| Poor glycemic control and normal arterial stiffness | 1.144(0.579-2.261) | 0.698 | 1.011(0.466-2.195) | 0.978 |
| Poor glycemic control and moderate arterial stiffness | 1.874(1.051-3.342) | 0.033 | 1.864(0.966-3.597) | 0.063 |
| Poor glycemic control and severe arterial stiffness ^a^ | 2.649(1.418-4.948) | 0.002 | 2.901(1.392-6.046) | 0.004 |
| Non-hypertension and normal arterial stiffness | ref |  | ref |  |
| Non-hypertension and moderate arterial stiffness | 1.503(0.942-2.399) | 0.088 | 1.433(0.886-2.317) | 0.143 |
| Non-hypertension and severe arterial stiffness ^a^ | 1.841(1.036-3.272) | 0.038 | 1.931(1.074-3.471) | 0.028 |
| Hypertension and normal arterial stiffness | 0.978(0.457-2.093) | 0.955 | 0.891(0.402-1.974) | 0.776 |
| Hypertension and moderate arterial stiffness ^a^ | 1.806(1.124-2.903) | 0.015 | 1.909(1.176-3.1) | 0.009 |
| Hypertension and severe arterial stiffness ^a^ | 2.642(1.591-4.386) | <0.001 | 2.685(1.597-4.514) | < 0.001 |

Normal arterial stiffness (baPWV <1400 cm/s); moderate arterial stiffness (1400≤ baPWV <1800 cm/s); severe arterial stiffness (baPWV ≥1800 cm/s).

Model 1 was adjusted for age and sex; model 2 was further adjusted for obesity, eGFR, LDL cholesterol, systolic pressure (if not stratified), fasting glucose (if not stratified), current smoking, and physical activity.

^a^ indicates P value <0.05.

**Table S5:** Association of arterial stiffness and alternatively defined glycemic control status with macrovascular complications.

|  | Model 1 | | Model 2 | |
| --- | --- | --- | --- | --- |
|  | HR (95% CI) | P value | HR (95% CI) | P value |
| Good glycemic control and normal arterial stiffness | Ref |  | Ref |  |
| Good glycemic control and moderate arterial stiffness | 2.35(0.89-6.21) | 0.085 | 2.34(0.88-6.20) | 0.089 |
| Good glycemic control and severe arterial stiffness ^a^ | 3.73(1.38-10.14) | 0.010 | 3.58(1.30-9.81) | 0.013 |
| Poor glycemic control and normal arterial stiffness | 1.93(0.73-5.09) | 0.184 | 1.93(0.73-5.15) | 0.187 |
| Poor glycemic control and moderate arterial stiffness ^a^ | 2.96(1.17-7.47) | 0.022 | 2.92(1.15-7.42) | 0.025 |
| Poor glycemic control and severe arterial stiffness ^a^ | 4.12(1.59-10.72) | 0.004 | 4.01(1.53-10.53) | 0.005 |

Normal arterial stiffness (baPWV <1400 cm/s); moderate arterial stiffness (1400≤ baPWV <1800 cm/s); severe arterial stiffness (baPWV ≥1800 cm/s).

Poor glycemic control was defined as fasting glucose ≥ 5.6 mmol/L.

Model 1: adjusted for age (continuous) and sex; Model 2: adjusted for age (continuous), sex, obesity (yes/no), eGFR (continuous), LDL cholesterol (continuous), hypertension (yes/no), current smoking (yes/no), and physical activity (yes/no).

Abbreviations: baPWV, brachial-ankle pulse wave velocity; LDL, low-density lipoprotein; eGFR, estimated glomerular filtration rate.

^a^ indicates P value <0.05.

**Table S6:** Association of arterial stiffness and alternatively defined hypertension status with macrovascular complications.

|  | Model 1 | | Model 2 | |
| --- | --- | --- | --- | --- |
|  | Hazard Ratio (95% CI) | P value | HR (95% CI) | P value |
| Non-hypertension and normal arterial stiffness | Ref |  | Ref |  |
| Non-hypertension and moderate arterial stiffness | 1.50(0.94-2.40) | 0.088 | 1.42(0.88-2.29) | 0.148 |
| Non-hypertension and severe arterial stiffness ^a^ | 1.84(1.04-3.27) | 0.037 | 1.92(1.08-3.43) | 0.027 |
| Hypertension and normal arterial stiffness | 0.98(0.46-2.09) | 0.955 | 0.90(0.41-1.97) | 0.784 |
| Hypertension and moderate arterial stiffness ^a^ | 1.81(1.12-2.90) | 0.015 | 1.93(1.19-3.12) | 0.008 |
| Hypertension and severe arterial stiffness ^a^ | 2.64(1.59-4.39) | <0.001 | 2.69(1.61-4.50) | <0.001 |

NHPNAS indicates no hypertension with normal arterial stiffness (baPWV <1400 cm/s); NHPMAS, no hypertension with moderately elevated arterial stiffness (1400≤ baPWV <1800 cm/s); NHPSAS, no hypertension with severely elevated arterial stiffness (baPWV ≥1800 cm/s); HPNAS, hypertension with normal arterial stiffness; HPMAS, hypertension with moderately elevated arterial stiffness; HPSAS, hypertension with severely elevated arterial stiffness.

Hypertension was defined as systolic blood pressure ≥130 mmHg or diastolic blood pressure ≥80 mmHg, the use of any antihypertensive medication, or self-reported diagnosis history of hypertension.

Model 1: adjusted for age (continuous) and sex; Model 2: adjusted for age (continuous), sex, obesity (yes/no), eGFR (continuous), LDL cholesterol (continuous), glycemic control(good/poor), current smoking (yes/no), and physical activity (yes/no).

Abbreviations: baPWV, brachial-ankle pulse wave velocity; LDL, low-density lipoprotein; eGFR, estimated glomerular filtration rate.

^a^ indicates P value <0.05.

**Table S7:** Association of per-SD increase of baPWV with diabetic macrovascular complication stratified by glycemic control and hypertension.

|  | Model 1 | | P _for interaction_ | Model 2 | | P _for interaction_ |
| --- | --- | --- | --- | --- | --- | --- |
|  | baPWV (per-SD) | P value |  | baPWV (per-SD) | P value |  |
| Glycemic control |  |  |  |  |  |  |
| good | 1.159(0.973-1.38) | 0.148 | 0.441 | 1.13(0.937-1.363) | 0.200 | 0.424 |
| poor ^a^ | 1.527(1.219-1.914) | <0.001 |  | 1.428(1.191-1.712) | <0.001 |  |
| Hypertension |  |  |  |  |  |  |
| no | 1.189(0.96-1.473) | 0.112 | 0.787 | 1.27(0.997-1.618) | 0.053 | 0.933 |
| yes ^a^ | 1.265(1.076-1.487) | 0.004 |  | 1.28(1.084-1.512) | 0.004 |  |

Poor glycemic control is defined as fasting glucose ≥ 7.0 mmol/L.

Hypertension was defined as systolic blood pressure ≥140 mmHg or diastolic blood pressure ≥90 mmHg, the use of any antihypertensive medication, or self-reported diagnosis history of hypertension.

Model 1 was adjusted for age and sex; model 2 was further adjusted for obesity, eGFR, LDL cholesterol, hypertension (if not stratified), glycemic control (if not stratified), current smoking, and physical activity.

Abbreviations: BaPWV, brachial-ankle pulse wave velocity.

**Table S8:** The composition of arterial stiffness, glycemic control and hypertension in each scored population.

|  | Overall | 0 | 1 | 2 | 3 | 4 |
| --- | --- | --- | --- | --- | --- | --- |
| Participants, No. | 1870 | 122 | 486 | 644 | 481 | 137 |
| Good glycemic control (n, %) | 844 (45.1) | 122 (100.0) | 257 (52.9) | 278 (43.2) | 187 (38.9) | 0 (0.0) |
| Poor glycemic control (n, %) | 1026 (54.9) | 0 (0.0) | 229 (47.1) | 366 (56.8) | 294 (61.1) | 137 (100.0) |
| Non-hypertension (n, %) | 1005 (53.7) | 122 (100.0) | 425 (87.4) | 372 (57.8) | 86 (17.9) | 0 (0.0) |
| Hypertension (n, %) | 865 (46.3) | 0 (0.0) | 61 (12.6) | 272 (42.2) | 395 (82.1) | 137 (100.0) |
| BaPWV (n, %) |  |  |  |  |  |  |
| <1400 cm/s | 465 (24.9) | 122 (100.0) | 290 (59.7) | 53 ( 8.2) | 0 (0.0) | 0 (0.0) |
| 1400-1799 cm/s | 936 (50.1) | 0 (0.0) | 196 (40.3) | 532 (82.6) | 208 (43.2) | 0 (0.0) |
| ≥1800 cm/s | 469 (25.1) | 0 (0.0) | 0 (0.0) | 59 ( 9.2) | 273 (56.8) | 137 (100.0) |

Poor glycemic control is defined as fasting glucose ≥ 7.0 mmol/L.

Hypertension was defined as systolic blood pressure ≥140 mmHg or diastolic blood pressure ≥90 mmHg, the use of any antihypertensive medication, or self-reported diagnosis history of hypertension.

Abbreviations: BaPWV, brachial-ankle pulse wave velocity.

**Table S9:** Association of weighted score with diabetic macrovascular complications.

|  | Hazard Ratio (95% CI) | P value |
| --- | --- | --- |
| Weighted score (per-unit increase) |  |  |
| Model 1 | 1.232 (1.109-1.397) | <0.001 |
| Model 2 | 1.214 (1.124-1.431) | <0.001 |

Model 1 was adjusted for age (continuous) and sex; model 2 was further adjusted for obesity (yes/no), eGFR (continuous), LDL cholesterol (continuous), current smoking (yes/no) and physical activity (yes/no).

**Additional Figures**


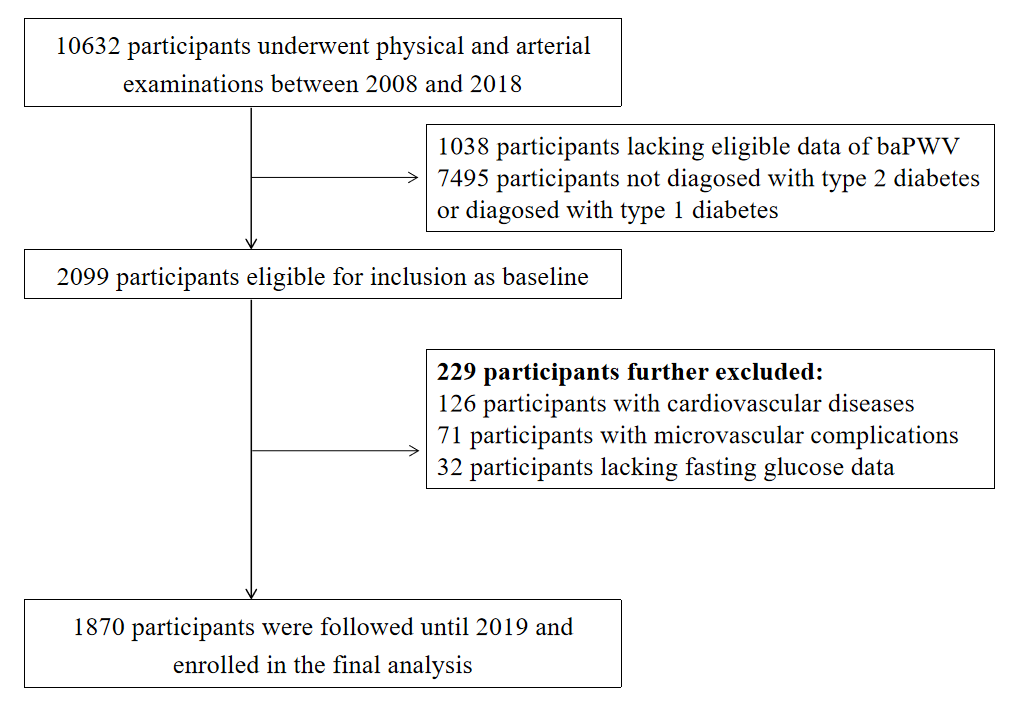


**Figure S1:** Flowchart of this current study.

**
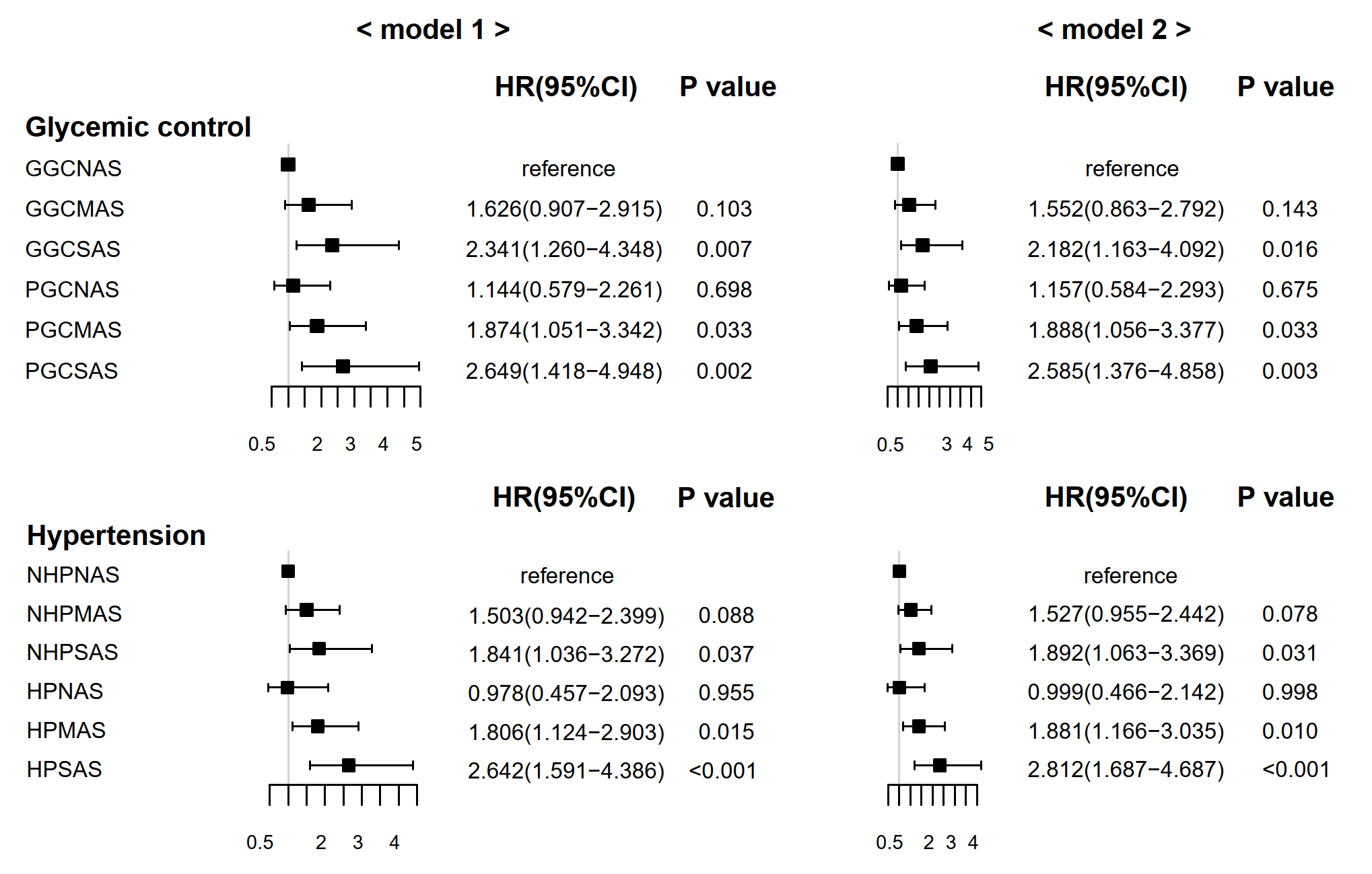
**

**Figure S2:** Association of arterial stiffness, glycemic control and hypertension status with development of macrovascular complication in imputed data.

GGCNAS represents good glycemic control with normal arterial stiffness (baPWV <1400 cm/s); GGCMAS, good glycemic control with moderately elevated arterial stiffness (1400≤ baPWV <1800 cm/s); GGCSAS, good glycemic control with severely elevated arterial stiffness (baPWV ≥1800 cm/s); PGCNAS, poor glycemic control with normal arterial stiffness; PGCMAS, poor glycemic control with moderately elevated arterial stiffness; PGCSAS, poor glycemic control with severely elevated arterial stiffness.

NHPNAS indicates no hypertension with normal arterial stiffness (baPWV <1400 cm/s); NHPMAS, no hypertension with moderately elevated arterial stiffness (1400≤ baPWV <1800 cm/s); NHPSAS, no hypertension with severely elevated arterial stiffness (baPWV ≥1800 cm/s); HPNAS, hypertension with normal arterial stiffness; HPMAS, hypertension with moderately elevated arterial stiffness; HPSAS, hypertension with severely elevated arterial stiffness.
